# Nuclear Receptor Nr4a1 Regulates Striatal Striosome Development and Dopamine D_1_ Receptor Signaling

**DOI:** 10.1523/ENEURO.0305-19.2019

**Published:** 2019-10-09

**Authors:** Maria-Daniela Cirnaru, Chiara Melis, Tomas Fanutza, Swati Naphade, Kizito-Tshitoko Tshilenge, Brian S. Muntean, Kirill A. Martemyanov, Joshua L. Plotkin, Lisa M. Ellerby, Michelle E. Ehrlich

**Affiliations:** 1 Department of Neurology, Icahn School of Medicine at Mount Sinai, New York, New York 10029; 2 The Buck Institute for Research on Aging, Novato, California 94945; 3 Department of Neuroscience, The Scripps Research Institute, Jupiter, Florida 33458; 4 Department of Neurobiology and Behavior, Stony Brook University, Stony Brook, New York 11794

**Keywords:** dopamine receptor D_1_, ERK, Nr4a1, signal, striosome, transduction

## Abstract

The GABAergic medium-size spiny neuron (MSN), the striatal output neuron, may be classified into striosome, also known as patch, and matrix, based on neurochemical differences between the two compartments. At this time, little is known regarding the regulation of the development of the two compartments. *Nr4a1*, primarily described as a nuclear receptor/immediate early gene involved in the homeostasis of the dopaminergic system, is a striosomal marker. Using *Nr4a1*-overexpressing and *Nr4a1*-null mice, we sought to determine whether Nr4a1 is necessary and/or sufficient for striosome development. We report that *in vivo* and *in vitro*, *Nr4a1* and *Oprm1* mRNA levels are correlated. In the absence of Nr4a, there is a decrease in the percentage of striatal surface area occupied by striosomes. Alterations in *Nr4a1* expression leads to dysregulation of multiple mRNAs of members of the dopamine receptor D_1_ signal transduction system. Constitutive overexpression of *Nr4a1* decreases both the induction of phosphorylation of ERK after a single cocaine exposure and locomotor sensitization following chronic cocaine exposure. *Nr4a1* overexpression increases MSN excitability but reduces MSN long-term potentiation. In the resting state, type 5 adenylyl cyclase (AC5) activity is normal, but the ability of AC5 to be activated by Drd1 G-protein-coupled receptor inputs is decreased. Our results support a role for *Nr4a1* in determination of striatal patch/matrix structure and in regulation of dopaminoceptive neuronal function.

## Significance Statement

This study provides insight into the role of *Nr4a1*, a transcription factor belonging to the family of orphan nuclear receptors *Nurr*, in the development of the striatal striosomal compartment and in the regulation of dopaminoceptive neuronal function. We show that the alteration of *Nr4a1* expression impacts the expression of striosome markers *in vivo* and *in vitro*, suggesting that Nr4a1 is necessary for full striosome development. Moreover, *Nr4a1* overexpression alters Drd1 signal transduction at multiple levels, resulting in reduced phosphorylation of ERK after cocaine administration, reduced induction of LTP, and the absence of locomotor sensitization following chronic cocaine use. These results indicate that the pathways regulated by *Nr4a1* may represent novel, druggable approaches to pathologic states such as levodopa-induced dyskinesia and cocaine sensitization.

## Introduction

The dorsal striatum is a subcortical nucleus with a role in the regulation of movement, reward, and cognition. More than 90% of the striatal neurons are GABAergic medium-sized spiny projecting neurons (MSNs) and are dopaminoceptive. They are subclassified as direct MSNs (dMSNs), expressing the dopamine (DA) D_1_ receptor (D_1_R) and projecting to the substantia nigra (SN), or indirect MSNs (iMSNs), expressing the dopamine D_2_ receptor and projecting to the globus pallidus. In addition, MSNs may be divided into patch (i.e. striosomes) or matrix compartments (Crittenden and Graybiel, 2011; Brimblecombe and Cragg, 2017). The striosomes comprise 10–15% of the striatal volume, receive limbic inputs, and contain both direct and indirect MSNs, with current data indicating a preponderance of dMSNs (Miyamoto et al., 2018). The transcription factor Nr4a1, called Nurr77, is an orphan member of the *Nurr* family of steroid/thyroid-like receptors (Giguère, 1999), appears as early as embryonic day 14.5 (E14.5) in the mouse, and marks striosomal MSNs (Davis and Puhl, 2011). Other striosomal markers include the μ-opioid receptor 1 [*Oprm1*/MOR (μ-opioid receptors)], substance P (*Tac1*), and calretinin fibers. Matrix MSNs express calbindin (*Calb1*), somatostatin (*Sst*), and enkephalin (*Penk*; Crittenden and Graybiel, 2011).

*Nr4a1* is expressed in dopaminergic and dopaminoceptive neurons, including in the dorsal striatum, nucleus accumbens, olfactory tubercle, and prefrontal and cingulate cortex (Zetterström et al., 1996; Beaudry et al., 2000; Werme et al., 2000a); and at lower levels, in SN and ventral tegmental area (VTA). Dopamine receptor antagonists, psychostimulants, or DA denervation induce the expression of *Nr4a1* in the midbrain dopaminergic SN and VTA and increase its expression in the striatum, where it acts as an immediate early gene (IEG; Beaudry et al., 2000; Werme et al., 2000a,b; St-Hilaire et al., 2003a; Ethier et al., 2004). Murine *Nr4a1* genetic deletion is associated with an increase in tyrosine hydroxylase, dopamine turnover (Gilbert et al., 2006), baseline locomotor activity (Gilbert et al., 2006; Rouillard et al., 2018), and tardive dyskinesia (Ethier et al., 2004), but a reduction in levodopa induces dyskinesia [levodopa-induced dyskinesia (LID)] in both rodent and nonhuman primate models of Parkinson’s disease (St-Hilaire et al., 2003a,b; Mahmoudi et al., 2009, 2013).

We began our studies in the *Nr4a1*-eGFP mouse (#GY139Gsat/Mmucd, GENSAT) to assay dopamine signal transduction in the striosomes, but found that the responses of these mice to dopamine agonists differed from those of nontransgenic littermates. We determined that the baseline *Nr4a1* mRNA level in this line is twice the wild-type (WT) level. Herein, comparing the *Nr4a1*-eGFP mouse to the previously characterized *Nr4a1*-null mouse (Lee et al., 1995), we sought to determine the role of *Nr4a1* in striosome development and regulation of the physiology of MSNs, and the dopamine signal transduction pathway. Our data indicate that Nr4a1 is necessary for, and promotes, the complete maturation of the striosome compartment, and its constitutive overexpression alters the D_1_R signaling pathway and response to cocaine.

## Materials and Methods

### Animals

Animal procedures were conducted in accordance with the NIH *Guidelines for the Care and Use of Experimental Animals* and were approved by the Institutional Animal Care Committee. The *Nr4a1-*eGFP hemizygous, *Nr4a1-*null (JAX Nr4a1tm1Jm #006187), *Drd1*-eGFP [Tg(Drd1a-eGFP)X60Gsat], and *Drd1* tdTomato (catalog #016204, The Jackson Laboratory) mice used for this study were obtained from GENSAT and The Jackson Laboratory, respectively. Controls always consisted of wild-type littermates. Mice were given *ad libitum* access to food and water and housed under a 12 h light/dark cycle. Only male mice were used in these studies.

### Drugs and treatment

Cocaine (20 mg/kg, i.p.) and MK-801 (0.1 mg/kg, i.p.; Sigma-Aldrich) were dissolved in 0.9% (w/v) NaCl (saline). Mice were habituated to handling and saline injection for 3 consecutive days before the experiment. Drugs were administered on day 4. MK-801 was administered 30 min before the cocaine injection.

### Locomotor activity

Locomotor activity was measured using the Digiscan D-Micropro automated activity monitoring system (Accuscan), consisting of transparent plastic boxes (45 × 20 × 20) set inside metal frames that were equipped with 16 infrared light emitters and detectors with 16 parallel infrared photocell beams. The breaks were recorded by a computer interface in 5 min bins. Mice were injected with cocaine (20 mg/kg, i.p.) or saline (3 ml/kg, i.p.) for 5 consecutive days. On days 1 and 5, mice were placed into the boxes and activity was recorded for 60 min during acclimation to the chamber. After 60 min, cocaine or saline was administered, and mice were immediately returned to the boxes for an additional 60 min of recording.

### Tissue preparation and immunofluorescence

At 4 months, mice were anesthetized with pentobarbital (30 mg/kg, i.p.) and transcardially perfused with ice-cold 0.1 m PBS, pH 7.5 (PBS 1×) and 4% (w/v) paraformaldehyde (PFA). Brains were postfixed overnight in the same solution and stored at 4°C in PBS. The 30 µm serial coronal free-floating sections were cut on a Leica vibratome, collected in cryobuffer (30% ethylene glycol, 20% glycerol in PBS 1×, pH 7.4) and stored at 4°C.

Postnatal day 3 (P3) mice were rapidly killed by decapitation, and brains were removed, washed in ice-cold PBS, and postfixed for 24 h at 4°C in 4% PFA. The brains were then incubated in 30% sucrose/1× PBS for 24 h at 4°C and cryopreserved in OCT embedding medium (catalog #4583, Tissue-Tek, Sakura). Serial coronal section (16 µm) were cut on a Leica cryostat, collected on Superfrost Plus Microscope Slides (Thermo Fisher Scientific), and frozen at −20°C.

Immunofluorescence was performed as previously described (Keilani et al., 2012). Sections were incubated with rabbit anti-phospho-p44/42 MAPK (ERK 1/2; Thr202/Tyr204; 1:500; catalog #9101S, Cell Signaling Technology), goat anti-c-*fos* (1:500, catalog #sc-52-G, Santa Cruz Biotechnology), rabbit anti-DARPP-32 (1:250; catalog #2306S, Cell Signaling Technology), mouse anti-calbindin 1 (1:500; catalog #C9848-2ml, Millipore-Sigma), or rabbit anti-MOR (1:1000; catalog #24216, Immunostar) antibodies. The respective secondary antibodies used were as follows: anti-mouse Alexa Fluor 488 (1:400; catalog #A-11008, Thermo Fisher Scientific), anti-mouse Alexa Fluor 594 (1:400; catalog #A-11005, Thermo Fisher Scientific), anti-rabbit Alexa Fluor 488 (1:400; catalog #A-11034, Thermo Fisher Scientific), or anti-rabbit Alexa Fluor 594 (1:400; catalog #A-11012, Thermo Fisher Scientific). Sections were sealed with Vectashield hard set mounting medium (catalog #H-1400, Vector Laboratories). Images were acquired using an Olympus BX61 epifluorescence microscope or a Panoramic 250 digital scanner (3DHISTECH) using a motorized 40× objective and a fluorescent camera. Quantification for the microscopy experiments was performed using slide scanner images except for the quantification of the striosomal area and the number for which both BX61 and slide scanner images were used.

### Striosome quantification

Striosome number and area, as a percentage of total striatal area, were measured in coronal sections from either matched adult or P3 mice immunolabeled with anti-MOR or anti DARPP-32, respectively. Using Fiji (version 2.0.0), the images were set at identical thresholds, and the regions of interest (ROIs) were outlined by manual tracing and managed with ROI manager function. The area and number of the ROIs selected were calculated using the Fiji “measure” function.

### Cell counting

The induction of phosphorylated ERK (pERK) differs by region, so the numbers of pERK and c-*fos* cells were counted specifically in the dorsomedial area of the striatum at rostral +1.18 mm, relative to bregma, and at caudal 0.86 mm. Cells were quantified in a fixed area using CaseViewer software by an observer blinded to treatment.

To determine the number and percentage of Drd1, *Nr4a1*-eGFP, and Drd2 cells in striosomes, we used coronal sections from double-hemizygous *Nr4a1*-eGFP/Drd1-Tomato (Shuen et al., 2008) at bregma 0.86 mm. The striosomal area was outlined by manual tracing, and the cells were counted as GFP^+^, Tomato^+^, and GFP/Tomato^+^. The percentage of each population was calculated relative to the total number of cells indicated by 4[prime],6[prime]-diamidino-2-phenylindole dihydrochloride (DAPI) staining.

### Primary neuronal cultures

*Nr4a1*-eGFP hemizygous and WT mice were timed mated, and the striatum was removed from E16.5 embryos by microdissection in cold Invitrogen Leibovitz’s medium (L-15, Thermo Fisher Scientific). The tissue was incubated in Ca^2+^/Mg^2+^-free HBSS for 10 min at 37°C. The incubation mixture was replaced with 0.1 mg/ml papain in Hibernate E/Ca^2+^ (BrainBits), incubated for 8 min, and rinsed in DMEM with 20% fetal bovine serum and twice in Leibovitz’s medium (L-15). The tissue was then suspended in DMEM with 10% fetal calf serum, glucose (6 mg/ml), glutamine (1.4 mm), and penicillin/streptomycin (100 U/ml). Cells were triturated through a glass-bore pipette and plated onto either Lab Tek eight-well slides (125,000 cells/well) for immunocytochemistry or 24-well plates (1 × 10^6^ cells/well) for RT-PCR analysis, each previously coated with polymerized polyornithine (0.1 mg/ml in 15 mm borate buffer, pH 8.4) and air dried. One hour later, the media were replaced with Invitrogen Neurobasal/B27 medium (Thermo Fisher Scientific) with GLUTAMAX and penicillin/streptomycin, and select wells were treated with brain-derived neurotrophic factor (BDNF; catalog #248-BD, R&D Systems) 25 ng/ml in 0.1% BSA/1× PBS. Media change and BDNF treatment were performed every 2 d, and the cells were kept in culture until day *in vitro* 7 (DIV7).

### *Nr4a1*-eGFP adenovirus transduction

Adenovirus (ADV)-CMV-*Nr4a1*-eGFP and ADV-CMV-eGFP were produced by SignaGen Laboratories. *Nr4a1*-eGFP ADV was produced using the human *Nr4a1* cDNA sequence. Viral transduction was performed after cells had attached for 48 h with a multiplicity of infection (MOI) of 20. The virus was added in fresh medium, and the medium was changed 18 h later. Cells were harvested or fixed 96 h following the addition of virus. ADV transduction in primary neurons was performed in four independent sets of cultures.

### Human induced pluripotent stem cell-derived NSC culture, differentiation, and transduction

Human induced pluripotent stem cells (iPSCs) were differentiated into prepatterned Activin A-treated neural stem cells (NSCs) using the following protocol. Briefly, iPSC colonies were detached using 1 mg/ml collagenase in Gibco KnockOut DMEM/F-12 medium (Thermo Fisher Scientific), and the resulting cell clumps were transferred to a 0.1% agarose-coated low-attachment Petri dish in embryonic stem (ES) culture medium [Gibco KnockOut DMEM/F12 (Thermo Fisher Scientific) supplemented with 20% Gibco KnockOut Serum Replacement (Thermo Fisher Scientific), 2.5 mm l-glutamine, 1× Non-Essential Amino Acids (NEAAs), 15 mm HEPES, 0.1 mm β-mercaptoethanol, 100 U/ml penicillin, 100 μg/ml streptomycin]. Every 2 d, 25% of ES medium was replaced by embryoid body (EB) differentiation medium (DMEM supplemented with 20% FBS, 1× NEAA, 2 mm l-glutamine, 100 U/ml penicillin, and 100 μg/ml streptomycin). At day 8, 100% of the culture medium was EB medium. At day 10, the embryoid bodies were attached to dishes coated with poly-l-ornithine (1:1000 in PBS; catalog #P3655, Sigma-Aldrich) and laminin (1:100 in KnockOut DMEM/F-12; catalog #L2020, Sigma-Aldrich), and cultured in neural induction medium [DMEM/F12 supplemented with 1× N2 (Thermo Fisher Scientific), 100 U/ml penicillin, and 100 μg/ml streptomycin] and 25 ng/ml bFGF (Peprotech) and 25 ng/ml Activin A (Peprotech). Media change was performed every 2 d. Rosettes were harvested after 7–10 s, plated on poly-l-ornithine- and laminin-coated dishes, and cultured in Neural Proliferation Medium (NPM; Neurobasal medium, 1× Gibco B27-supplement [Thermo Fisher Scientific), 2 mm l-glutamine, 10 ng/ml leukemia inhibitory factor (catalog #300-05, Peprotech), 100 U/ml penicillin, 100 μg/ml streptomycin] supplemented with 25 ng/ml bFGF and 25 ng/ml Activin A. The resulting NSCs were passaged and maintained in this same medium. These prepatterned Activin A-treated NSCs were validated by immunofluorescence analysis and labeled positively for putative NSC markers, namely Nestin, SOX1, SOX2, and PAX6.

For the ADV transduction experiments human iPSC-derived NSCs were plated at 100,000 cells per well of a 6-well plate in 2 ml NPM supplemented with 10 ng/ml FGF2 (Peprotech) and 10 ng/ml Activin A (Peprotech). At 70% confluency, they were transduced with ADV-CMV-NR4A1-eGFP or ADV-CMV-eGFP at MOI of 20 suspended in 2 ml NPM without penicillin/streptomycin antibiotic. Nontransduced NSCs and NSCs transduced with ADV-eGFP were used as controls. A complete media change was performed 24 h post-transduction. Cells were harvested 14 d after transduction for gene expression and immunolabeling assays. ADV transduction in a human iPSC-derived NSC culture was performed twice, with three replicates each.

### Cell immunofluorescence

Cultures were fixed in 4% paraformaldehyde in 0.1 m phosphate buffer, pH 7.4, and immunolabeled with rabbit anti-DARPP-32 (1:500; catalog #2306S, Cell Signaling Technology) followed by anti-rabbit Alexa Fluor 594 (1:400; catalog #A-11012, Thermo Fisher Scientific). To identify the total number of cells, the nuclei were stained with DAPI (1:10,000; Millipore-Sigma). Images were acquired using an Olympus BX61 microscope and were analyzed using Fiji software (ImageJ).

### Quantitative real-time PCR

Snap-frozen samples for gene expression assays were homogenized in QIAzol Lysis Reagent (Qiagen). Total RNA was extracted with the miRNeasy Mini Kit (Qiagen) according to the manufacturer instructions. RNAs, 500 ng, were reversed transcribed using the High Capacity RNA-to-cDNA Kit (Applied Biosystems). Quantitative real-time PCR (qRT-PCR) was performed in a Step-One Plus System (Applied Biosystems) using All-in-One qPCR Mix (GeneCopoeia).

For qRT-PCR analysis of prepatterned Activin A-treated human NSCs, total RNA was isolated using the ISOLATE II RNA Mini Kit (Bioline). cDNA was prepared from 1 μg of RNA in a total reaction volume of 20 μl using the SensiFAST cDNA synthesis kit (Bioline). RT-PCR reactions were set up in a 384-well format using 2× SensiFAST Probe No-ROX Kit (Bioline) and 1 μl of cDNA per reaction in a total volume of 10 μl. RT-PCR was performed on the Roche LightCycler 480 instrument.

Quantitative PCR consisted of 40 cycles, 15 s at 95°C and 30 s at 60°C each, followed by dissociation curve analysis. The ΔCt was calculated by subtracting the Ct for the endogenous control gene GAPDH from the Ct of the gene of interest. Mouse and human primer sequences are listed in Table 1 and in Table 2, respectively. Relative quantification was performed using the ΔΔCt method (Livak and Schmittgen, 2001) and is expressed as a fold change relative to control by calculating 2^-ΔΔCt^.

**Table 1.** qRT-PCR primers sequence

Gene	Primer forward 5´-3´	Primer reverse 5´-3´
*Adcy5*	GGCAGCTGGAAAAGATCAAG	CCAGCCACTACAGGTCCAAT
*Calb 1*	ACTCTCAAACTAGCCGCTGCA	TCAGCGTCGAAATGAAGCC
*Rasgrp1*	GGACCTACCAAGAACTGGAA C	GATCCCAGTAAACCCGTCTG
*Ppp1r1b*	GAAGAAGAAGACAGCCAGGC	TAGTGTTGCTCTGCCTTCCA
*Drd1*	TTCTTCCTGGTATGGCTTGG	GCTTAGCCCTCACGTTCTTG
*Drd2*	TGGACTCAACAACACAGACCAGAATG	GATATAGACCAGCAGGTTGACGATGA
GAPDH	AACGACCCCTTCATTGACCT	TGGAAGATGGTGATGGGCTT
*Foxp2*	AAGCAGCTTGCCTTTGCTAAG	GGATTGAATGTATGTGTGGCTGA
*Oprm1*	CCCTCTATTCTATCGTGTGTGT	AGAAGAGAGGATCCAGTTGCA
*Ppp1cc*	CATCGACAGCATCATCCAAC	GGAAAGCCACCGTATTCAAA
*Nr4a1*	ATGCCTCCCCTACCAATCTTC	CACCAGTTCCTGGAACTTGGA
*Nr4a2*	CAGCTCGAGCCACATAAACA	TCCTTGTCCGCTCTCTTCAT
*Ptpn5*	CTCTGGACCCTTTCTTGCTG	GGATCTTCAGGGTCTGGTGA

**Table 2.** qRT-PCR human primer sequences

Gene	Primer forward 5´-3´	Primer reverse 5´-3´
*Ppp1r1b*	CACACCACCTTCGCTGAAA	GAAGCTCCCCCAGCTCAT
*Oprm1*	AGAAACAGCAGGAGCTGTGG	ACCGAGACTTTTCGGGTTC
*Calb2*	GAAAATCGAGATGGCAGAGC	CATAAACTCGGCGCTGGA
*Calb1*	CACAGCCTCACAGTTTTTCG	CCTTTCCTTCCAGGTAACCA
*Bcl11b*	CCCAGAGGGAGCTCATCAC	GACACTGGCCACAGGTGAG
*ACTB*	CCAACCGCGAGAAGATGA	TCCATCACGATGCCAGTG

### Western blotting

Snap-frozen striatum samples dissected from *Nr4a1-*eGFP, *Nr4a1*-null, and WT mice were lysed in Pierce RIPA buffer (Thermo Fisher Scientific) containing freshly added Pierce phosphatase and protease inhibitors (Thermo Fisher Scientific). The supernatant was collected after centrifugation (20 min at 15,000 × *g* at 4°C), and the protein concentration was determined using the BCA method. For each sample, 30 μg of protein was resolved in 4–12% Bis/Tris-acrylamide gradient gels (Bio-Rad) and transferred to nitrocellulose membranes. The membranes were incubated with the following primary antibodies: rabbit anti-ERK1/2 (1:500; catalog #9102, Cell Signaling Technology), mouse anti-AC5 (type 5 adenylyl cyclase; Xie et al., 2015), and mouse anti-GAPDH (1:5000; catalog #sc32233, Santa Cruz Biotechnology) for the normalization. The following secondary antibodies were used: anti-rabbit HRP-conjugated (1:2000; catalog #PI-1000, Vector Laboratories) or anti-mouse HRP-conjugated (1:2000; catalog #PI-2000, Vector Laboratories). Following development with Pierce ECL (Thermo Fisher Scientific), pictures were acquired using a Fujifilm ImageReader LAS-4000, and bands were quantified using Fiji software (ImageJ).

### Patch-clamp recordings

Patch-clamp whole-cell recordings were performed from the dorsal striatum of the 300-µm-thick coronal brain slices obtained from 4-month-old mice. An upright microscope with differential interference contrast, fluorescence, and IR (Nikon Eclipse E600FN, Morrel Instrument) was used to visualize the neurons. The cells were voltage clamped at −70 mV, using patch pipettes (resistance, 3–6 MΩ) filled with an internal solution containing the following (in mm): 115 K-gluconate, 20 NaCl, 1.5 MgCl2, 10 phosphocreatine-Tris, 2 Mg-ATP, 0.5 Na-GTP, and 10 HEPES, pH 7.3 and 286 mmol/kg osmolarity. The hemi-slices were transferred to a recording chamber constantly perfused with oxygenated aCSF at a flow rate of ∼4 ml/min for gravity. Experiments were performed at 28.0 ± 0.1°C. Series resistance was monitored through the experiments, and cells with a >10% change in series resistance were excluded from analysis. Recordings were acquired with a Multiclamp 700B Amplifier (Molecular Devices) and Digidata 1440A digitizer (Molecular Devices). Current-clamp protocols were designed and performed using pClamp 10.3 Electrophysiology Data Acquisition and Analysis Software. Output signals were acquired at 5 kHz, filtered at 2.4 kHz, and stored on-line using pCLAMP 10.3 Electrophysiology Data Acquisition and Analysis Software (Molecular Devices). Single-cell long-term depression (LTD) was induced in the presence of 10 µm 1(S)9(R)(−) bicuculline methiodide using a high-frequency stimulation (HFS) protocol (Calabresi et al., 1997) consisting of four 1-s-duration, 100 Hz trains delivered at a frequency of one train every 10 s. Square-wave current pulses (60 µs pulse width) were delivered with a concentric bipolar electrode placed above the corpus callosum through a stimulus isolator (Isoflex, AMPI). Output signals were acquired at 5 kHz, filtered at 2.4 kHz, and stored on-line using pCLAM 10.3Electrophysiology Data Acquisition and Analysis Software (Molecular Devices). In all cases, the experimenter was blind to genotype and or/treatment.

### Field electrophysiology

Coronal brain slices containing the striatum were prepared from 9-week-old WT or *Nr4a1*-eGFP mice. Animals were anesthetized with isoflurane, and brains were rapidly removed from the skull and placed in ice-cold modified solution (aCSF) containing the following (in mm): 215 sucrose, 2.5 KCl, 1.6 NaH_2_PO_4_, 4 MgSO4, 1 CaCl2, 4 MgCl2, 20 glucose, and 26 NaHCO3, pH 7.4, and equilibrated with 95% O_2_ and 5% CO_2_. Coronal brain slices (250 µm thick) were prepared with a VT1000S Vibratome (Leica Microsystems), incubated at 31°C for 30 min, and then stored at room temperature for ≥1 h in normal aCSF containing the following (in mm): 120 NaCl, 3.3 KCl, 1.2 Na_2_HPO_4_, 26 NaHCO3, 1.3 MgSO4, 1.8 CaCl2, and 11 glucose, pH 7.4 equilibrated with 95% O_2_ and 5% CO_2_, 280–300 mmol/kg osmolarity. The hemi-slices were transferred to a recording chamber constantly perfused with oxygenated aCSF at a flow rate of ∼4 ml/min using a peristaltic pump (Masterflex C/L); experiments were performed at 28.0 ± 0.1°C. Recordings were acquired with a GeneClamp 500B Amplifier (Molecular Devices) and a Digidata 1440A digitizer (Molecular Devices). All signals were low-pass filtered at 2 kHz and digitized at 10 kHz. For extracellular field recordings (fEPSP recordings), a patch-type pipette was fabricated on a micropipette puller (Sutter Instrument), filled with normal aCSF (resistance, 3–6 MΩ), and placed in the dorsomedial striatum to measure long-term potentiation (LTP). A Concentric Bipolar Electrode stimulator (FHC) was placed immediately above the corpus callosum. Before and after HFS, the stimulus intensity was set to the level at which an evoked population spike was around half of the amplitude of the maximal obtainable response. Stimulus intensity was adjusted to a level evoking a maximal response during HFS. Stimulus intensity ranged from 0.3 to 1.2 mA (Partridge et al., 2000). Paired-pulse facilitation was measured by delivering two stimuli at 20, 50, and 100 ms interstimulus intervals before HFS. Each interstimulus interval was repeated three times, and the resulting potentials were averaged. LTP was induced using an HFS protocol, as follows: four 1 s duration, 100 Hz trains delivered at a frequency of one train every 10 s. Square-wave current pulses (60 µs pulse width) were delivered through a stimulus isolator (Isoflex, AMPI). Stimulus intensity was adjusted to evoke a maximal response during HFS (Partridge et al., 2000; Kreitzer and Malenka, 2007).

### cAMP measurement and adenylyl cyclase activity assay

Flash-frozen striatal tissue punches from adult *Nr4a1*-eGFP and WT mice were homogenized in 250 μl 0.1N HCl, centrifuged at 1000 × *g* for 15 min, and supernatants diluted 20-fold for cAMP quantification using a cAMP enzyme immunoassay kit (cAMP Direct EIA) following the acetylated protocol (Enzo). The activity of adenylyl cyclase (AC) in striatal membrane preparations was determined as described previously (Xie et al., 2012). Briefly, striatal tissue punches were flash frozen in liquid nitrogen before homogenization in a buffer containing the following (in mm): HEPES, pH 8.0 (20); EDTA (1); NaCl (150); MgCl_2_ (2), dithiothreitol (1); and 1× complete protease inhibitor cocktail (Roche). After centrifugation at 1000 × *g* for 15 min, the supernatant was subject to ultracentrifugation at 25,000 rpm for 35 min in a Beckman SW41 rotor over a 23%/43% sucrose gradient. The plasma membrane fraction was isolated from the sucrose interface, and the concentration was determined using the Pierce 660 nm Protein Assay Reagent (Thermo Fisher Scientific). Striatal membranes (2 μg/reaction) were treated with vehicle (basal) or indicated stimulator for 10 min at 30°C in AC assay buffer (50 mm HEPES, pH 8.0; 0.6 mm EDTA; 100 μg/ml BSA; 100 μm 3-isobutyl-1-methylxanthine; 3 mm phosphoenolpyruvate potassium; 10 μg/ml pyruvate kinase; 5 mm MgCl2; 10 µm GTP; and 100 μm ATP). Reactions were stopped by adding an equal volume of 0.2N HCl. The resulting cAMP in the sample was determined by cAMP Direct EIA kit.

### Statistics

Statistical analysis was performed using GraphPad software version 6. One-way ANOVA followed by Sidak’s multiple-comparisons test was performed for one-factor comparisons versus control. For 2 × 2 comparisons, two-way ANOVA was used with repeated measures for the appropriate factor followed by Bonferroni’s or Holm–Sidak’s *post hoc* comparisons. For the RT-PCR and Western blot densitometry, we performed one-way ANOVA with genotype factor analysis, followed by *post hoc* tests with multiple comparisons versus control or WT mice. To analyze locomotor data and AC5 activity results, two-way ANOVA with treatment and genotype factors were used. For the electrophysiology experiments, statistical comparisons of pooled data were performed by unpaired *t* test or ANOVA (one-way or two-way). Results were considered significant at *p* < 0.05. Values are presented as the mean ± SEM based on the number of samples that were used in each experiment.

## Results

### *Nr4a1* mRNA expression in *Nr4a1*-eGFP and *Nr4a1*-null mice

**Figure 1.**
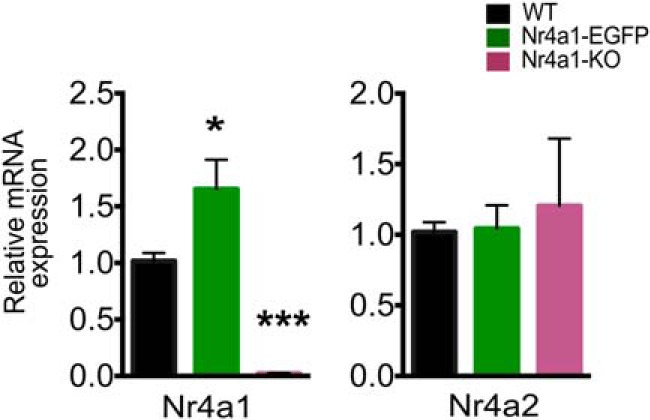
*Nr4a1* mRNA is increased in *Nr4a1*-eGFP mice, and *Nr4a2* mRNA is equal to wild type. qRT-PCR expression analysis of *Nr4a1* and *Nr4a2* in 4-month-old WT, *Nr4a1-*eGFP, and *Nr4a1*-null mice. *n* = 6 mice/genotype; one-way ANOVA corrected for multiple comparisons and Sidak’s post-test with genotype factor. For *Nr4a1* expression: *F*_(2,14)_ = 23.42, *p* < 0.0001; *Nr4a2* expression: *F*_(2,20)_ = 0.1871, *p* = 0.8308. *p = 0.0145, ***p = 0.0010. Data are presented as the mean ± SEM.

Some GENSAT mice created using BAC technology [e.g. *Drd2*-eGFP (Kramer et al., 2011) and *ChAT*-Cre (Chen et al., 2018)] show expression from the transgene, increasing the total expression level of the BAC-encoded gene. Similarly, *Nr4a1*-eGFP adult hemizygous mice express twice as much *Nr4a1* mRNA in the striatum relative to wild-type littermates (Fig.1; *F*_(2,14)_ = 23.42, *p* < 0.0001; Sidak’s multiple-comparisons test, *t*_(14)_ = 2.787, *p* = 0.0145). The exact cause of *Nr4a1* overexpression remains unknown. However, despite being engineered to prevent the increased levels of the reporter gene, several BAC mice show increased levels of the gene under study, or of other genes encoded on the BAC or impacted by insertion of the BAC (Kolisnyk et al., 2013; Ting and Feng, 2014). Confirming previous results (Lee et al., 1995), *Nr4a1* mRNA expression is abolished in *Nr4a1* homozygote-null [knock-out (KO)] mice (Fig.1; Sidak’s multiple-comparisons test, *t*_(14)_ = 4.166, *p* = 0.0010). The level of mRNA of a second *Nurr* family member, *Nr4a2*, mRNA levels is unchanged in both genotypes (Fig. 1; *F*_(2,20)_ = 0.1871, *p* = 0.8308). We were unable to confirm the specificity of commercially available anti-Nr4a1 antibodies for immunocytochemistry or Western blotting.

### *Nr4a1* striatal distribution and effect of its overexpression or deletion on striosome compartment maturation *in vivo*

We analyzed several aspects of striatal phenotype in *Nr4a1*-eGFP and *Nr4a1*-null mice, including the compartmentalization of EGFP^+^ neurons, the surface area occupied by striosomes; and the mRNA levels of striosome, matrix, and markers of common MSNs. *Ppp1r1b*/DARPP-32 served as an early marker of striosomes, and as a general MSN marker in adult striatum. *Calb1* (i.e. calbindin 28 kDa) was used as a marker of the matrix compartment, and *Oprm1*/MOR, *Rasgrp1*/Caldag-GEFII, and, to a lesser extent, *Foxp2*, as markers of striosomes in the adult (Crittenden et al., 2009; Crittenden and Graybiel, 2011).

**Figure 2.**
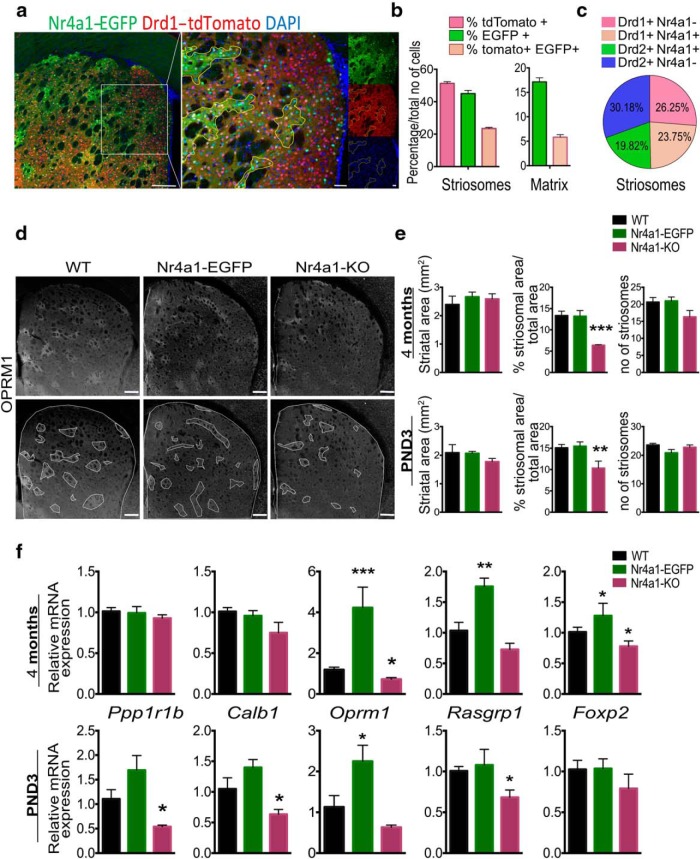
Constitutive upregulation or downregulation of *Nr4a1* mRNA alters spatial development of the striosomal compartment and mRNA levels of its markers *in vivo*. ***a***, Coronal section of adult *Nr4a1-*eGFP*/Drd1-*tdTomato showing the colocalization of Drd1-tdTomato and Nr4a1-eGFP. The ROI selection indicates the section represented in higher magnification. Single channels are shown in the miniatures. Scale bars: 200 and 50 µm. ***b***, Quantification of tdTomato^+^, EGFP^+^, and tdTomato^++^/EGFP^+^ cells in striosomes and matrix shown in ***a***. Percentage of each cell population was calculated relative to the total number of cells counted by DAPI immunofluorescence. ***c***, Graphic representation of the percentage distribution of Drd1^+^0/Nr4a1^−^, Drd1^+^/Nr4a1^+^, Drd2^+^/Nr4a1^+^, and Drd2^+^/Nr4a1^−^ in the striosomes. For this, the Drd1 (i.e., tdTomato^−^) cells were counted as Drd2^+^ cells. ***d***, Representative OPRM1 immunolabeling on 30-µm-thick coronal sections from 4-month-old WT, *Nr4a1-*eGFP, and *Nr4a1-*null mice with superimposition of selected ROIs delineating the total striatal and striosomal areas in the bottom panel. Scale bars, 200 µm. ***e***, Quantification of the striatal area, the percentage of the area occupied by the striosomes, and of the number of striosomes in 4-month-old and P3 WT, *Nr4a1-*eGFP, and *Nr4a1*-null mice shows a decrease in the percentage of the area occupied by the striosomes in *Nr4a1*-null mice at both ages. For both P3 and adult analysis, *n* = 6 mice/genotype. One-way ANOVA corrected for multiple comparisons (Sidak’s test). For adults: striatal area *F*_(2,15)_ = 1.897, *p* = 0.1943; striosomal area: *F*_(2,15)_ = 40.83, *p* < 0.0001; WT vs *Nr4a1-*eGFP: *t*_(15)_ = 0.1803, *p* = 0,8593; WT vs *Nr4a1-*null: *t*_(15)_ = 7.914, ***p = 0.0001; number of striosomes: *F*_(2,15)_ = 2.007, *p* = 0.1689. For P3: striatal area: *F*_(2,19)_ = 1,5481, *p* = 0.2383; striosomal area: *F*_(2,19)_ = 12.87, *p* = 0.0003; WT vs *Nr4a1-*eGFP: *t*_(19)_ = 0.3318, *p* = 0.7437; WT vs *Nr4a1*-KO: *t*_(19)_ = 4.333, **p = 0.0004; number of striosomes: *F*_(2,15)_ = 2.818, *p* = 0.0914. Data are presented as the mean ± SEM. ***f***, mRNA levels of striosome and matrix marker as determined by qRT-PCR of striatal mRNA from 4-month-old and P3 WT, *Nr4a1-*eGFP, and *Nr4a1*-null mice reveals a positive correlation between *Nr4a1* expression levels and striosomal markers *Oprm1* (*F*_(2,20)_ = 15.52, *p* < 0.0001; followed by Sidak’s multiple-comparisons test vs WT: *t*_(20)_ = 4.732, *p* = 0.0001 for *Nr4a1*-eGFP; *t*_(20)_ = 1.099, *p* = 0.2848 for Nr4a1-KO), *Rasgrp1* (*F*_(2,11)_ = 16.49, *p* = 0.0005; *t*_(11)_ = 4.082, *p* = 0.0018; *t*_(11)_ = 1.639, *p* = 0.1295), and *Foxp2* (*F*_(2,11)_ = 20.02, *p* = 0.0002; *t*_(11)_ = 2.795, *p* = 0.0174; *t*_(11)_ = 3.174, *p* = 0.0089) in the adult. In P3, Nr4a1-KO mice have reduced levels of *Ppp1r1b* (*F*_(2,15)_ = 13.96, *p* = 0.0004; *t*_(15)_ = 2.899, *p* = 0.011), *Calb1* (*F*_(2,12)_ = 10.77, *p* = 0.0021; *t*_(12)_ = 2.721, *p* = 0.0186), and *Rasgrp1* (*F*_(2,8)_ = 4.657, *p* = 0.0456; *t*_(8)_ = 2.378, *p* = 0.0447). *n* ≥ 5 mice/genotype, one-way ANOVA corrected for multiple comparisons (Sidak’s test). *p < 0.05, **p < 0.01, ***p < 0.001. Data are presented as the mean ± SEM.

We quantitated the distribution of Nr4a1-eGFP, dMSNs, and iMSNs in the two compartments, using double-hemizygous Nr4a1-eGFP/Drd1-tdTomato adult mice (Fig. 2*a*). In the striosomes, we counted a total of 506 cells, of which 50% were tdTomato^+^, 43% EGFP^+^, and 23% were double labeled. In the matrix, from a total of 1362 cells, only 17.1% were EGFP^+^ and only 5.8% were double labeled (Fig. 2*b*). Therefore, in the striosomes, assuming that the majority of Drd1-tdTomato^−^/DAPI^+^ cells are Drd2^+^, iMSNs and dMSNs are present at equal levels (i.e. 50% each). Focusing on Nr4a1 coexpression, 24% of the cells are Drd1^+^/Nr4a1^+^ and 20% are Drd2^+^/Nr4a1^+^ (Fig. 2*c*).

*Nr4a1* overexpression in the GENSAT mouse does not alter the general architecture of the striatum as the area occupied by the striosomes is unchanged from the WT mouse at both P3 and in the adult (Fig. 2*e*; Sidak’s multicomparisons test: adults, *t*_(15)_ = 0.1803, *p* = 0.8593; P3, *t*_(19)_ = 0.3318, *p* = 0.7437). *Nr4a1* deletion, however, reduces the area occupied by striosomes at both P3 and 4 months, as determined by Oprm1 immunostaining (Fig. 2*e*; P3: *t*_(19)_ = 4.333, *p* = 0.0004; adults: *t*_(15)_ = 7.914, *p* = 0.0001). The number of striosomes is unchanged (Fig. 2*e*; *F*_(2,15)_ = 2.007, *p* = 0.1689; and *F*_(2,15)_ = 2.818, *p* = 0.0914). There is a positive correlation between *Nr4a1* and *Oprm1* mRNA levels at both ages in the *Nr4a1-*eGFP mouse (Fig. 2*f*; *F*_(2, 20)_ = 15.52, *p* < 0.0001 and *F*_(2, 12)_ = 8.711, *p* = 0.0046, respectively). *Rasgrp1* mRNA level is increased at 4 months in the *Nr4a1*-eGFP mouse (Fig. 2*f*; Sidak’s multicomparisons test: *t*_(11)_ = 4.082, *p* = 0.0018), and, relative to the WT mouse, decreased in the *Nr4a1*-null mouse at P3 (Fig. 2*f*; *t*_(8)_ = 2.378, *p* = 0.0447) with a trend downward at 4 months (Fig. 2*f*; *t*_(11)_ = 1.639, *p* = 0.1295). *Calb1* mRNA level is decreased in the *Nr4a1*-null mouse at P3 (*t*_(12)_ = 2.721, *p* = 0.0186), but is normal in the adult (*t*_(11)_ = 1.377, *p* = 0.1969).

### Nr4a1 promotes maturation of the medium spiny neuron *in vitro*

**Figure 3.**
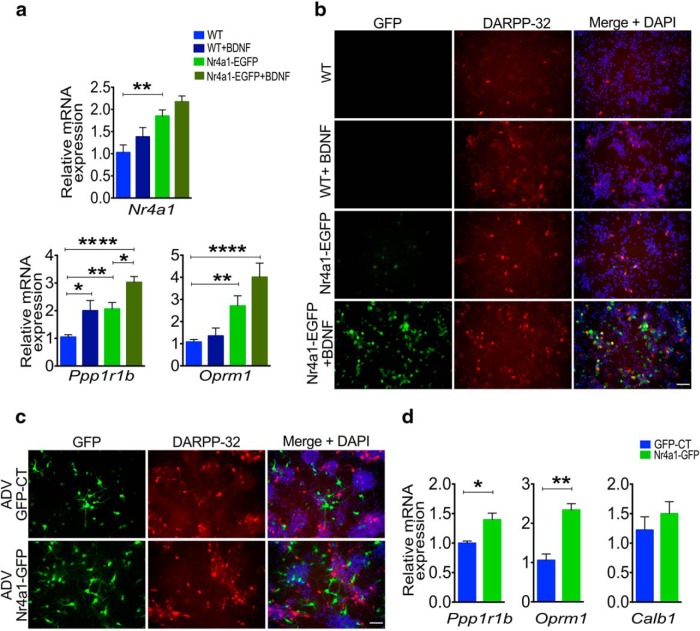
Nr4a1 promotes the maturation of specific medium spiny neuronal phenotypes, including of *Oprm1*, a striosomal marker. ***a***, qRT-PCR assay of *Nr4a1*, *Ppp1r1b*, and *Oprm1* mRNAs on DIV7 WT and *Nr4a1-*eGFP primary striatal neurons treated with BDNF 25 ng/ml vs 0.1% BSA reveals that *Nr4a1* is overexpressed in *Nr4a1-*eGFP neurons (*F*_(3,15)_ = 10.15, *p* = 0.007; WT vs *Nr4a1-*eGFP: *t*_(15)_ = 3695, *p* = 0.0022) and is associated with an increase in *Ppp1r1b* (*F*_(3,27)_ = 10.04, *p* = 0.0001; WT vs *Nr4a1-*eGFP: *t*_(27)_ = 2.910, *p* = 0.0072) and *Oprm1* (*F*_(3,13)_ = 17.77, *p* < 0.0001; WT vs *Nr4a1-*eGFP *t*_(13)_ = 3.338, *p* = 0.0053). BDNF treatment is additive for both *Ppp1r1b* (WT vs WT+BDNF: *t*_(27)_ = 2.729, *p* = 0.011; WT vs *Nr4a1-*eGFP+BDNF: *t*_(27)_ = 5.479, *p* = 0.0001; *Nr4a1-*eGFP vs *Nr4a1-*eGFP+BDNF: *t*_(27)_ = 2.688, *p* = 0.0127) and *Oprm1* (WT vs *Nr4a1-*eGFP+BDNF: *t*_(13)_ = 5.944, *p* = 0.0001). *n* = 5 samples/genotype. One-way ANOVA corrected for multiple comparisons (Sidak’s test): *p < 0.05, **p < 0.01, ****p < 0.0001. Data are presented as the mean ± SEM. ***b***, Representative *Pp1r1b*/DARPP-32 staining on DIV7 WT and *Nr4a1-*eGFP primary striatal neurons treated with BDNF (25 ng/ml) shows a relatively increased number of DARPP-32-immunopositive cells in *Nr4a1-*eGFP primary cultures. The effects of increased Nr4a1 and BDNF are additive. Scale bars, 50 µm. ***c***, Representative DARPP-32 immunolabeling of WT primary striatal neurons 96 h after transduction with ADV-GFP CT vs ADV-*Nr4a1*-eGFP showing increase in DARPP-32-immunopositive cells in the cultures overexpressing Nr4a1. Scale bars, 50 µm. ***d***, qRT-PCR assay shows increases in *Ppp1r1b* (*t*_(6)_ = 2.551, *p* = 0.0434) and *Oprm1* (*t*_(6)_ = 5.505, *p* = 0.0015) mRNA levels in wild-type primary striatal neurons 96 h after transduction with ADV-*Nr4a1*-GFP. *n* = 5 samples/treatment, two-tailed unpaired *t* test: *p < 0.05, **p < 0.01. Data are presented as the mean ± SEM.

Given that *Nr4a1* overexpression increases the striatal level of *Oprm1* mRNA relative to wild type *in vivo*, we investigated whether *Nr4a1* overexpression impacts the maturation of MSNs *in vitro*. Lateral ganglionic eminence primary neuronal cultures from E16.5 *Nr4a1-*eGFP embryos, compared with cultures from wild-type mice, have higher levels of *Ppp1r1b/*DARPP-32 (Fig.3*a*; Sidak’s multiple comparisons test, *t*_(27)_ = 2.910, *p* = 0.0072) and *Oprm1* mRNAs (Fig. 3*a*; *t*_(13)_ = 3.338, *p* = 0.0053). BDNF promotes the maturation of MSNs and requires Egr-1 (Keilani et al., 2012). We found that the BDNF and Nr4a1 effects on the induction of DARPP-32 are additive (Fig 3*a*; *t*_(27)_ = 5.479, *p* = 0.0001; Fig. 3*b*) and that BDNF does not induce *Nr4a1* mRNA (*t*_(15)_ = 1.448, *p* = 1.682), implying the use of alternate signal transduction pathways. Importantly, EGFP fluorescence was visible in these cultures at the time of plating and *Nr4a1* mRNA was already increased (Fig. 3*b*), indicating expression from the BAC transgene at this early age.

**Figure 4.**
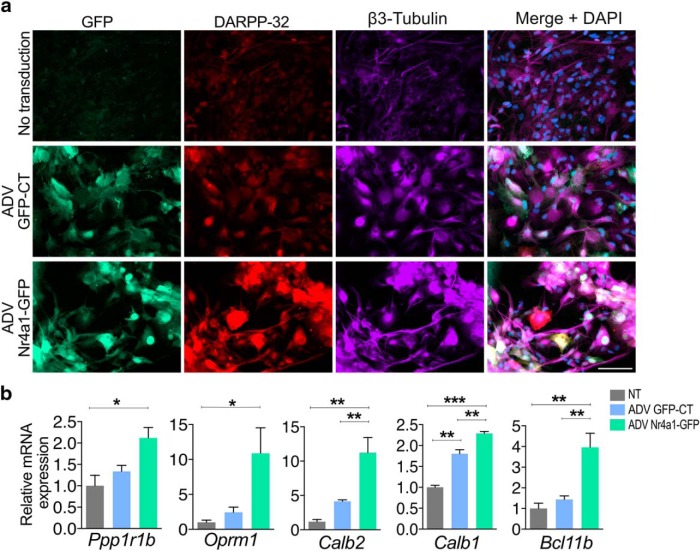
Nr4a1 promotes the differentiation of MSNs phenotypes in human iPSC-derived NPCs into medium spiny neurons. ***a***, Immunolabeling of DARPP-32 and βIII tubulin in iPSC-derived NSCs, untreated, vs transduction with ADV-GFP or ADV-NR4A1-GFP shows that Nr4a1 induces the upregulation of DARPP-32 and βIII tubulin. Scale bar, 100 µm. ***b***, qRT-PCR assays show an increase in *Ppp1r1b* (*F*_(2,6)_ = 7.173, *p* = 0.0256; NT vs Nr4a1-GFP: *t*_(6)_ = 3.691, *p* = 0.0102), *Oprm1* (*F*_(2,6)_ = 6.105, *p* = 0.0358; NT vs Nr4a1-GFP: *t*_(6)_ = 3.234, *p* = 0.0178), *Calb2* (*F*_(2,6)_ = 15.75, *p* = 0.0041; NT vs Nr4a1-GFP: *t*_(6)_ = 5.461, *p* = 0.0016; GFP-CT vs Nr4a1-GFP: *t*_(6)_ = 3.854, *p* = 0.008), *Calb1* (*F*_(2,5)_ = 72.94, *p* = 0.0002; NT vs Nr4a1-GFP: *t*_(5)_ = 11.63, *p* = 0.0001; GFP vs Nr4a1-GFP: *t*_(5)_ = 4.357, *p* = 0.0073), and *Bcl11b* (*F*_(2,6)_ = 13.69, *p* = 0.0058; NT vs Nr4a1-GFP: *t*_(6)_ = 4.848, *p* = 0.0029; GFP vs Nr4a1-GFP: *t*_(6)_ = 4.132, *p* = 0.0068) mRNA levels in human iPSC-derived NSCs transduced with ADV-Nr4a1-GFP for 14 d. *n* = 3 samples for each group. One-way ANOVA corrected for multiple comparisons (Tukey’s test): *p < 0.05, **p < 0.01, ***p < 0.001. Data are presented as the mean ± SEM.

We also transduced E16.5 wild-type MSNs *in vitro* with adenovirus driving expression from the human *Nr4a1* cDNA (ADV-CMV-Nr4a1). Similar to constitutive overexpression, exogenous Nr4a1 induced higher levels of *Ppp1r1b* (Fig. 3 *c*,*d*; unpaired two-tailed *t* test, *t*_(6)_ = 2.551, *p* = 0.0434) and *Oprm1* mRNAs (Fig. 3*d*; *t*_(6)_ = 5.505, *p* = 0.0015) compared with transduction with ADV-CMV-GFP (Fig. 3 *c*,*d*). Finally, we assayed the ability of Nr4a1 to induce the expression of MSN markers in human iPSC-derived neuronal precursors. For this, we analyzed the expression of striatal markers in ADV-CMV-Nr4a1- versus ADV-CMV-GFP-transduced human iNSCs at 14 d of differentiation. Nr4a1 induced the expression of neuronal marker β3 tubulin (Fig. 4*a*), of immature striosome-specific marker DARPP-32 (*Ppp1r1b*; *t*_(6)_ = 3.691, *p* = 0.0102), and of *OPRM1* (*t*_(6)_ = 3.234, *p* = 0.0178) and calretinin (*Calb2*; *t*_(6)_ = 5.461, *p* = 0.0016; Fig. 4*b*). Moreover, Nr4a1 overexpression also induced calbindin (*Calb1*; *t*_(5)_ = 11.63, *p* = 0.0001) and (*Bcl11b*; *t*_(6)_ = 4.848, *p* = 0.0029), indicating that in this system Nr4a1 promotes the differentiation of NSCs toward both the MSN striosome and matrix phenotypes.

### *Nr4a1* overexpression reduces induction of pERK and c-Fos after acute cocaine exposure and impairs locomotor sensitization to chronic cocaine

**Figure 5.**
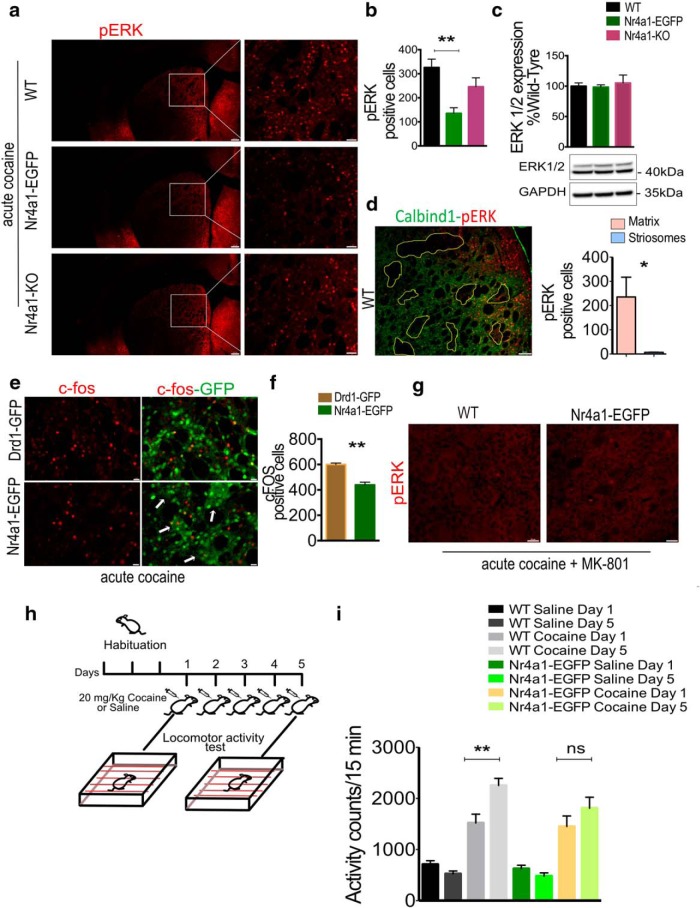
*Nr4a1* overexpression reduces the induction of phosphorylation of ERK and c-*fos* after acute cocaine injection and impairs locomotor sensitization to chronic cocaine. ***a***, Representative pERK immunolabeling indicating the dorsomedial region of interest and fixed area used for the quantification of pERK^+^ cells in the striatum of 4-month-old WT, *Nr4a1-*eGFP, and *Nr4a1*-null mice 10 min after a single intraperitoneal injection of cocaine (20 mg/kg). Scale bars: 200 and 50 µm. ***b***, Quantification of ***a*** showing a relative reduced induction of pERK^+^ cells in *Nr4a1-*eGFP mice (*F*_(2,8)_ = 7.281, *p* = 0.0158; WT vs *Nr4a1*-eGFP: *t*_(8)_ = 3.816, *p* = 0.0051). *n* = 4 mice/genotype; one-way ANOVA corrected for multiple comparisons (Bonferroni’s correction): *p < 0.05. Data are presented as the mean ± SEM. ***c***, ERK 1/2 basal protein levels are equal in 4-month-old WT, *Nr4a1-*eGFP, and *Nr4a1*-null mice (*F*_(2,9)_ = 1.340, *p* = 0.3094). *n* = 8 mice/genotype. One-way ANOVA corrected for multiple comparisons (Sidak’s test). Data are presented as the mean ± SEM. ***d***, Calbindin and pERK immunolabeling shows that the induction of pERK occurs predominantly in the matrix compartment after a single intraperitoneal injection of cocaine (20 mg/kg). Scale bars, 100 µm. The graph shows the quantification of pERK^+^ cells in matrix and striosomes in sections from bregma 0.86 mm. *n* =3 mice, unpaired *t* test: *t*_(2.003)_ = 4.702, *p = 0.0423. Data are presented as the mean ± SEM. ***e***, c-*fos* and GFP immunolabeling in the dorsal striatum of *Drd1-e*GFP and *Nr4a1-*eGFP adult mice 1 h after a single intraperitoneal injection of cocaine (20 mg/kg), indicating relatively reduced c-*fos* induction in *Nr4a1-*eGFP mice. Arrows indicate c-*fos* labeling in the GFP^+^ striosomes in the *Nr4a1-*eGFP mice. Scale bars, 50 µm. ***f***, Quantification of c-*fos* ^+^ cells in *Drd1*-eGFP and *Nr4a1-*eGFP adult mice shown in ***e***. *n* = 3 mice/genotype and treatment, unpaired *t* test: *t*_(4)_ = 6.721, **p = 0.0026. Data are presented as the mean ± SEM. ***g***, pERK immunostaining in WT and *Nr4a1-*eGFP mice after the injection NMDA antagonist MK-801(0.1 mg/kg) followed 30 min later by a single injection of cocaine (20 mg/kg) shows the abolition of pERK induction. Scale bars, 50 µm. ***h***, Schematic representation of treatments used for the induction of locomotor sensitization to cocaine. ***i***, *Nr4a1-*eGFP mice show decreased locomotor sensitization to chronic cocaine use relative to WT mice. One-way ANOVA corrected for multiple comparisons (Bonferroni’s correction; *F*_(7,108)_ = 8.639, *p* < 0.0001; WT cocaine day 1 vs WT cocaine day 5: *t*_(108)_ = 3.705, **p = 0.003; *Nr4a1-*eGFP cocaine day 1 vs *Nr4a1-*eGFP cocaine day 5: *t*_(108)_ = 0.6920, *p* = 0.4904). *n* = 15 mice/genotype, One-way ANOVA corrected for multiple comparisons (Bonferroni’s correction). Data are presented as the mean ± SEM. ns = non-significant.

LID induction following dopamine denervation of the striatum correlates with a high level of pERK (Santini et al., 2007; Westin et al., 2007; Alcacer et al., 2012; Cerovic et al., 2015) and requires a specific subpopulation of dMSNs (Girasole et al., 2018). Strikingly, LID is reduced in the absence of Nr4a1 (Rouillard et al., 2018), raising the question of whether Nr4a1 level regulates acute induction of pERK. Relative to WT, the overexpression or deletion of *Nr4a1* both decreased the induction of pERK 10 min after intraperitoneal cocaine administration, although the decrease was not significant in the null mouse (Fig. 5*a*,*b*; *F*_(2,8)_ = 7.281, *p* = 0.0158; Bonferroni’s multiple-comparisons test: *Nr4a1-*eGFP mice: *t*_(8)_ = 3.816, *p* = 0.0051; *Nr4a1*-null mice: *t*_(8)_ = 1.726, *p* = 0.1226). The number of pERK^+^ cells in saline-injected mice ranged from 7 to 10 in each genotype, similar to what is shown in the study by Valjent et al. (2004). Total ERK levels were unaltered (Fig. 5*c*; *F*_(2,9)_ = 1.340, *p* = 0.3094). Comparisons among nontransgenic (WT), *Drd1-*eGFP, and *Nr4a1-*eGFP mice demonstrated equal pERK induction in WT versus Drd1-eGFP mice (*F*_(2,9)_ = 1.584, *p* = 0.2574; data not shown). The majority of pERK^+^ cells were located in the matrix (Fig. 5*d*), both in WT and *Nr4a1*-EGFP mice.

Additional experiments were restricted to the *Nr4a1*-eGFP line. In *Nr4a1*-eGFP mice, c-*fos* induction 1 h after cocaine administration was reduced relative to *Drd1*-eGFP and appeared to occur primarily in the striosomal compartment (Fig. 5*e*,*f*; two-tailed unpaired *t* test, *t*_(4)_ = 6.721, *p* = 0.0026). Dopamine-mediated induction of pERK requires simultaneous activation of the D_1_ and NMDA receptors (Valjent et al., 2005; Girault et al., 2007; Girault, 2012a,b). As in WT animals, pretreatment with MK-801 almost entirely abolished the induction of pERK in *Nr4a1-*eGFP mice (Fig. 5*g*).

Induction of pERK and c*-fos* after a single exposure to cocaine correlates with locomotor sensitization induced after chronic cocaine exposure, but not with acute locomotor activity (Valjent et al., 2006; Xu, 2008). To assay acute locomotor response and sensitization, mice were injected for 5 d with cocaine (20 mg/kg, i.p.) or saline, and their locomotor activity was recorded for 60 min after the injections on days 1 and 5 (Fig. 5*h*; *F*_(7,108)_ = 8.639, *p* < 0.0001). *Nr4a1* overexpression did not alter the basal activity (Bonferroni’s multiple-comparisons test, *t*_(108)_ = 0.4087, *p* = 0.6836) or the locomotor response to the first injection of cocaine (*t*_(108)_ = 0.3489, *p* = 0.7278), but, contrary to WT mice (*t*_(108)_ = 3.705, *p* = 0.0003), the *Nr4a1-*eGFP mice did not sensitize (*t*_(108)_ = 0.6920, *p* = 0.4904; i.e. did not show an increase in their locomotor response to cocaine on day 5 relative to day 1; Fig. 5*i*) while presenting normal locomotor activity without any sign of confined stereotypy due to repeat exposure to cocaine.

### Impact of *Nr4a1* overexpression on electrophysiological properties of dorsomedial striosomal MSNs and on striatal synaptic plasticity

**Figure 6.**
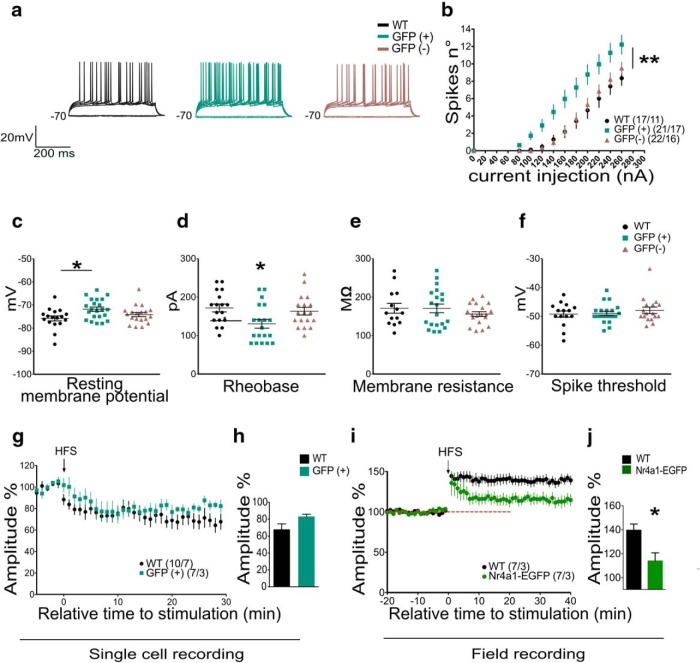
Characterization of electrophysiological properties of dorsomedial striosomal MSNs and the impact of Nr4a1 overexpression on striatal synaptic excitability and plasticity. ***a***, Action potential sample traces from single cells derived from the dorsal striatum in WT mice and in the center of the striosomes for GFP^+^ and GFP^–^ neurons from *Nr4a1*-eGFP mice. ***b***, Number of action potentials as a function of injected current intensity in WT, GFP^+^, and GFP^–^ neurons indicate that neuronal action potentials are increased in GFP^+^ neurons. Two-way ANOVA with genotype factor (*F*_(2,57)_ = 7.421, *p* = 0.0014). *n* ≥ 17/genotype and cell type; **p < 0.01. Data are presented as the mean ± SEM. ***c***, Resting membrane potential is more depolarized in GFP^+^ neurons compared with WT (WT MSNs: average, −75.8 mV, *n* = 17 cells; GFP^+^ MSNs: average, −71.7mV, *n* = 22; GFP^−^ MSNs: average, −74.2 mV, *n* = 21). One-way ANOVA followed by Bonferroni’s multiple-comparisons test: *F*_(2,55)_ = 4.303, *p* = 0.0183, *p < 0.05. Data are presented as the mean ± SEM. ***d***, Rheobase current is lower in GFP^+^ neurons relative to WT and GFP^−^ MSNs (WT MSNs: average, 171.1 pA, *n* = 17 cells; GFP^+^ MSNs: average, 134.1 pA, *n* = 22; GFP^−^ MSNs: average, 164.7 pA, *n* = 21). One-way ANOVA-Bonferroni’s multiple-comparison test, *F*_(2,51)_ = 4.342, *p* = 0.0181; *p < 0.05. Data presented as ± SEM. ***e***, Membrane resistance recorded in voltage-clamp experiments is equal in the three cell types. WT, *n* = 10; GFP^+^ and GFP^−^, *n* = 16. One-way ANOVA-Bonferroni’s multiple-comparison test: *F*_(2,50)_ = 0.7388, *p* = 0.4828. Data are presented as the mean ± SEM. ***f***, Spike threshold is equal in the three cell types. WT, *n* = 15; GFP^+^ and GFP^−^, *n* = 19. One-way ANOVA-Bonferroni’s multiple-comparison test: *F*_(2,49)_ = 0.5219, *p* = 0.5967. Data are presented as the mean ± SEM. ***g***, Whole-cell patch-clamp recordings of long-term synaptic depression (LTD) induced by high-frequency stimulation in WT and GFP^+^ MSNs showing overlapping traces for both genotypes. Data are presented as the mean ± SEM. ***h***, Bar graph representing the average of the last 5 min after LTD induction in WT and GFP^+^ MSNs indicating no significant differences between the two groups: WT, 10 recordings/7 mice; GFP^+^, 7 recordings/3 mice. Unpaired two-tailed *t* test, *t*_(15)_ = 1.868, *p* = 0.0815. Data are presented as the mean ± SEM. ***i***, LTP assayed in field recordings in WT and *Nr4a1-*eGFP shows reduced LTP in *Nr4a1-*eGFP mice after high-frequency stimulation. Data are presented as the mean ± SEM. ***j***, Bar graph representing the average of the last 5 min after LTP induction in field recordings (7 recordings and 3 mice for both genotypes). Unpaired two-tailed *t* test: *t*_(12)_ = 3.011, *p* = 0.0108; *p < 0.05; Data are presented as the mean ± SEM.

Altered dopaminergic transmission at corticostriatal synapses is associated with impaired bidirectional synaptic plasticity, including LTP, LTD, and depotentiation (Shen et al., 2008; Cerovic et al., 2015; Trusel et al., 2015). Specifically, ERK has a crucial role in LTP induction as ERK inhibitors attenuate or even eliminate LTP in dorsomedial striatum (Xie et al., 2009). In *Nr4a1*-EGFP mice, we performed single-cell patch clamp recordings of EGFP^+^ and EGFP^−^ neurons located in the center of striosomes. Based on the percentages of EGFP^+^ neurons that are also Drd1^+^ ( Fig. 2*a–c*), we estimated that half of the EGFP^+^ cells from which we recorded were dMSNs and half were iMSNs. In the WT mice, in which MSN subtypes are indistinguishable morphologically, we assumed that the majority of the neurons from which we recorded are located in the matrix, which represents ∼90% of the striatum, with a 1:1 distribution between dMSNs and iMSNs. Overall, EGFP^+^ neurons were more excitable than those recorded in WT mice, as determined by left-shifted current–frequency plots and lower rheobase currents (Fig. 6*a*,*b*; two-way ANOVA with genotype factor, *F*_(2,57)_ = 7.421, *p* = 0.0014). This might be due to an intrinsic increased excitability of striosomal neurons compared with matrix (Crittenden et al., 2017). Membrane resistance and spike threshold were equal in WT, and EGFP^+^ and EGFP^−^ MSNs (*F*_(2,50)_ = 0.7388, *p* = 0.4828 and *F*_(2,49)_ = 0.5219, *p* = 0.5967), but resting membrane potential (rheobase) was more depolarized in EGFP^+^ neurons (*F*_(2,55)_ = 4.303, *p* = 0.0183; Fig. 6*c–f*). Notably, we observed the difference in excitability in mixed populations of MSNs despite the fact that D_1_ dMSNs are less excitable than D_2_ iMSNs (Gertler et al., 2008; Planert et al., 2010) and the EGFP^+^ neurons are equally likely to be D_1_ or D_2_.

Using a standard high-frequency stimulation protocol (Calabresi et al., 1997), we observed that LTD induction was equivalent in WT neurons (cells registered from wild-type mice) and EGFP^+^ neurons (Fig. 6*g*,*h*; unpaired two-tailed *t* test, *t*_(15)_ = 1.868, *p* = 0.0815). LTP, however, could not be induced in the majority of EGFP^+^ neurons (only two of seven) using the same protocol, whereas it was readily induced in five of seven WT MSNs (data not shown). As we were unable to reliably obtain LTP in EGFP^+^ neurons with whole single-cell recordings, we also used field recordings to confirm this genotype-dependent effect, and assayed LTP, LTD, and paired-pulse ratio. In field recordings, LTD and paired-pulse ratio in *Nr4a1-*eGFP striatum were equivalent to those in WT (two-way ANOVA with genotype factor, *F*_(1,19)_ = 0.2400, *p* = 0.6298; data not shown), but LTP, albeit present, was significantly decreased in amplitude (Fig. 6*i*,*j*; unpaired two-tailed *t* test, *t*_(12)_ = 3.011, *p* = 0.0108). The normal paired-pulse ratio suggests that the corticostriatal glutamate release and AMPA receptor function are unaltered by Nr4a1 overexpression. The differences in LTP induction observed both by single-cell and field recordings strongly suggest an alteration in the activation of MSNs by constitutive overexpression of Nr4a1, but these data do not allow us to distinguish between the effects of genotype and compartment.

### Nr4a1 overexpression impacts the expression of Drd1 signaling pathway components

**Figure 7.**
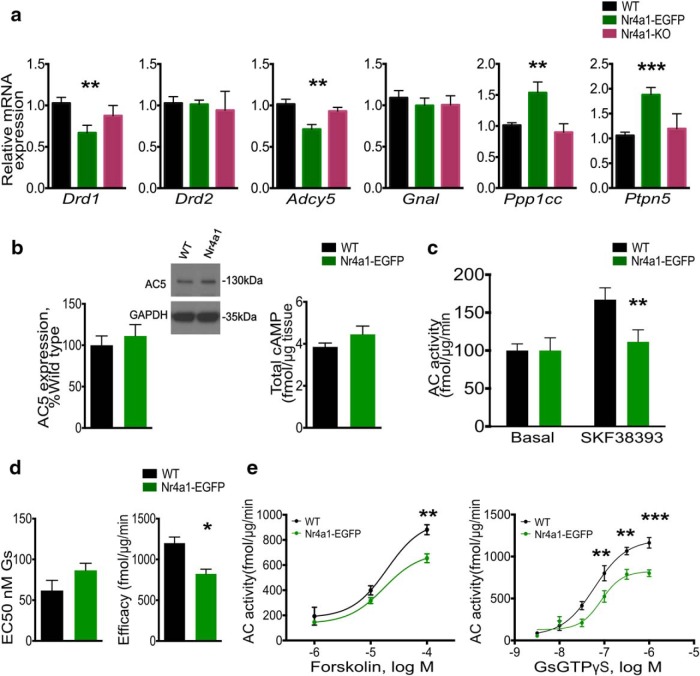
Nr4a1 overexpression impacts the activation of Drd1signaling. ***a***, qRT-PCR assay of mRNA levels of Drd1 signaling pathway components in the striatum of 4-month-old WT, *Nr4a1-*eGFP, and *Nr4a1*-null mice shows a decrease of *Drd1* (*F*_(2,21)_ = 4.755, *p* = 0.0198; *t*_(21)_ = 3.084, *p* = 0.0056) and *Adyc5*, and an increase of *Ppp1cc* (*F*_(2,24)_ = 7.275, *p* = 0.0034; *t*_(24)_ = 3.301, *p* = 0.003) and *Ptpn5* (*F*_(2,24)_ = 10.15, *p* = 0.0006; *t*_(24)_ = 4332, *p* = 0.0002) in *Nr4a1-*eGFP mice. *n* ≥ 5 mice/genotype; One-way ANOVA corrected for multiple comparisons (Sidak’s test): *p < 0.05, **p < 0.01, ***p < 0.001. Data are presented as the mean ± SEM. ***b***, Western blot of AC5 protein shows equal levels in WT and *Nr4a1*-eGFP striatum, normalized to GAPDH. Unpaired *t* test, *t*_(7)_ = 0.6399, *p* = 0.5426; *n* ≥ 4/genotype. Data are presented as the mean ± SEM. The basal striatal cAMP level in *Nr4a1*-eGFP mice is equal to wild type. Unpaired *t* test, *t*_(8)_ = 1.367, *p* = 0.2089. *n* ≥ 4/genotype. Data are presented as the mean ± SEM. ***c***, AC5 activity on Drd1 stimulation with SKF38393 indicates a reduction of the response in *Nr4a1-*eGFP mice. *n* = 3 mice/genotype. Two-way ANOVA corrected for multiple comparisons (Sidak’s test) *F*_(1,4)_ = 25.96, *p* = 0.007, *t*_(8)_ = 4.659, **p = 0.0016. Data are presented as the mean ± SEM. ***d***, E50 and AC5 stimulation efficacy in striatal membrane preparation from Nr4a1-eGFP and wild-type mice, showing a decrease in AC5 efficacy in Nr4a1-eGFP mice. *n* = 3 mice; unpaired *t* test, *t*_(4)_ = 3.484, *p = 0.0253. Data are presented as the mean ± SEM. ***e***, Gs-GTPγS and Forskolin AC5 activity–titration curves in striatal membrane preparation from *Nr4a1*-eGFP and wild-type mice, indicating a reduced AC5 activation plateau in Nr4a1-eGFP mice. *n* = 3 mice. Two-way ANOVA corrected for multiple comparisons (Sidak’s test) genotype factor forskolin: *F*_(1,12)_ = 12.76, *p* = 0.0038; at 10 mm: *t*_(12)_ = 3.941, *p* = 0.002; Gαs-GTPγS: *F*_(1,24)_ = 35.19, *p* < 0.0001; at 0.1 and 0.5 μm: *t*_(24)_ = 2.711, **p = 0.0122; and 1 μm: *t*_(24)_ = 3650, ***p = 0.0013. Data are presented as the mean ± SEM.

Dopamine induces pERK via a signal transduction pathway consisting of Drd1, Gα_olf_, and AC5, the activation of which results in production of the second messenger cAMP. The downstream effectors include cAMP-dependent protein kinase, DARPP-32, protein phosphatase-1 (PP1), and striatal-enriched tyrosine phosphatase 61 (STEP61; Girault, 2012a). We performed additional qPCR assays of genes in the Drd1 pathway and biochemical assays to assess its function. We identified a decrease in mRNA levels of *Drd1* (*F*_(2,21)_ = 4.755, *p* = 0.0198; and *t*_(21)_ = 3.084, *p* = 0.0056) and *Adcy5* (AC5; *F*_(2,24)_ = 7.618, *p* = 0.0027; and *t*_(24)_ = 3.859, *p* = 0.008), and an increase in *Ptpn5* (STEP61; *F*_(2,24)_ = 10.15, *p* = 0.0006; and *t*_(24)_ = 4332, *p* = 0.0002) and *Ppp1c* (PP1; *F*_(2,24)_ = 7.275, *p* = 0.0034; and *t*_(24)_ = 3.301, *p* = 0.003; Fig. 7*a*) in *Nr4a1*-eGFP mice relative to WT littermates. *Gnal* (Gα_olf_) mRNA was equal in all three genotypes (*F*_(2,15)_ = 0.3226, *p* = 0.7291; Fig 7*a*). Despite the decrease in AC5 mRNA, the baseline cAMP level was equal to WT (unpaired *t* test, *t*_(8)_ = 1.367, *p* = 0.2089) and, consistent with this finding, the level of AC5 protein was normal (unpaired *t* test, *t*_(7)_ = 0.6399, *p* = 0.5426), indicating post-transcriptional regulation (Fig. 7*b*). However, the ability of AC5 to be activated by Drd1 stimulation was diminished (Fig. 7*c*; *F*_(1,4)_ = 25.96, *p* = 0.007; and *t*_(8)_ = 4.659, *p* = 0.0016), as evidenced also by the reduced efficacy of cAMP response to dopamine stimulation (Fig. 7*d*; unpaired *t* test, *t*_(4)_ = 3.484, *p* = 0.0253). This suggests that the pool of activatable AC5 is diminished.

We next used forskolin and Gαs-GTPγS titrations to bypass Drd1-Gα_olf_ inputs and assay only the AC5 activation step. These experiments revealed that *Nr4a1-*eGFP mice have reduced the potency of AC activation compared with WT mice at equal dosage (Fig.7*e*; two-way ANOVA genotype factor; Forskolin: *F*_(1,12)_ = 12.76, *p* = 0.0038; Gαs-GTPγS: *F*_(1,24)_ = 35.19, *p* < 0.0001), suggesting that the regulation of AC5 by sensitizing/desensitizing factors is abnormal.

## Discussion

Nr4a1 is a member of the Nur family of nuclear receptors, which are expressed in specific patterns in the CNS and periphery. In the brain, a high level of Nr4a1/Nur77 is found in dopaminoceptive striatal MSNs, where it is enriched in the striosomes (Davis and Puhl, 2011). The *Nr4a1*-null mouse has been characterized to some extent (Gilbert et al., 2006), but its striosomal architecture has not been described, and the effects of *Nr4a1* overexpression have not been reported. In this study, we show that the GENSAT *Nr4a1-*eGFP reporter mouse expresses twice the normal level of *Nr4a1* mRNA in the striatum, allowing us to examine the effects of *Nr4a1* overexpression and deletion on specific aspects of striatal development and function. However, the mechanism leading to Nr4a1 overexpression in this animal model remains unknown.

We found that in the presence of increased Nr4a1, several markers of striosomal MSNs are increased both in the early postnatal period and in the adult, whereas markers of matrix MSNs are largely unchanged. Although striosomes are clearly demarcated in the absence of *Nr4a1*, they are smaller and occupy a lower percentage of the total area. Moreover, Nr4a1 overexpression in human-derived NSCs differentiated with Activin A further promotes their maturation toward a general MSN phenotype. Notably, Nr4a1 promotes maturation of the medium spiny neuron *in vitro*, including several striosome markers. The exact mechanism via which Nr4a1 regulates striosome formation is unknown, but a microarray study of hippocampal neurons in which Nr4a1 is overexpressed revealed the upregulation of several transcription factors also involved in striatal development, including Sp8, Meis1, and Gsx1 (Chen et al., 2014). We conclude that a wild-type level of Nr4a1 is required for normal striosome development and maintenance, suggesting unique functions of Nr4a1 and the absence of compensation by other members of the Nur family.

*Nr4a1* deletion alters striatal response to dopamine agonists and antagonists, and the data herein show that overexpression leads to dysregulation of striatal plasticity and response to external stimuli. It remains to be determined how much of this is due to constitutive overexpression and/or the increased induction of *Nr4a1* as an IEG due to the BAC transgene. Notably, the acute effects may be mitigated to some extent by the decreased induction of pERK, which is required for the induction of *Nr4a1* transcription (Bourhis et al., 2008; Yue et al., 2017). Genetic disruption of *Nr4a1* in rats has recently been shown to reduce the development of LIDs (Rouillard et al., 2018), but pERK level was not assayed in these animals. We are attempting to distinguish between the effects of these opposing activities on ERK phosphorylation.

Focusing on direct pathway function, we found that *Nr4a1* overexpression impairs Drd1 signaling, reducing cocaine-induced phosphorylation of ERK, locomotor sensitization to cocaine, and LTP. We found dysregulation of multiple components of this signal transduction pathway that may contribute to decreased Drd1 signaling (Girault et al., 2007; Girault, 2012a,b), but not all in the same direction, making it difficult to attribute any changes to one specific molecule. Overall, we conclude that *Nr4a1* overexpression compromises AC5 availability reducing the efficacy of its responses to stimulatory GPCR inputs. As AC5 is the predominant cyclase in MSNs (Lee et al., 2002), it is unlikely that decreased AC activation reflects the dysfunction of an alternate cyclase. Interestingly, an increase in G_iα_ inputs could reduce AC5 activation, and Oprm1, which is increased in *Nr4a1-*eGFP MSNs, is a G_iα_ coupled receptor (Chakrabarti et al., 1995; Saidak et al., 2006; Lamberts et al., 2011; Traynor, 2012). Activation of AC5 via Oprm1 is required for morphine-induced locomotor activity (Kim et al., 2006). Therefore, chronically increased MOR tone could impact cyclase activation. Likewise, with our current knowledge, we are unable to pinpoint the etiology of ERK dysregulation. Increased activity of PP1 could decrease ERK phosphorylation (for review, see Pascoli et al., 2014), as could a genetic, albeit compensatory, increase in STEP61, as constitutive deletion of *STEP* leads to an increase in pERK1/2 levels (García-Forn et al., 2018). Conversely, CalDEG-GEFII (called Rasgrp1) is increased in striosomes in a rat model of LIDs, and its dysregulation in the presence of an increase in Nr4a1 may also contribute to the regulation of ERK phosphorylation (Crittenden et al., 2009), but the possible increase in activity of this pathway due to increased *Nr4a1* clearly does not overcome whatever is inhibiting the phosphorylation of ERK.

Not surprisingly, the motor and signal transduction abnormalities in the *Nr4a1-*eGFP mice are associated with striatal electrophysiological abnormalities, which require further investigation. The decreased induction of pERK, the apparent reduction in LTP, and the lack of locomotor sensitization to cocaine are internally consistent. Changes in excitability may contribute to alteration in networks and plasticity, thereby impacting the response to psychostimulant (for review, see Crittenden and Graybiel, 2011; Cao et al., 2018). How striosomes may impact movement and response to psychostimulants remains an open question that should be further studied using methods in which striosomes and Drd1 are delineated in the absence of any molecular changes, so that effects of both compartmentalization and MSN subtypes can be distinguished. The D_1_R proportion of striosomes is highly dependent on their location and the relative expression of Oprm1 and the neuropeptides SP and ENK (Tajima and Fukuda, 2013; Miyamoto et al., 2018), the latter of which corresponds with the distribution of Nr4a1. Here we concluded that *Nr4a1*-EGFP is expressed equivalently in dMSNs and iMSNs, whereas Davis and Puhl (2011) reported an enrichment of Nr4a1-EGFP in the dMSNs. This discrepancy may arise from their use of Drd1 immunolabeling to identify dMSNs and in the location of the striosomes.

The composite effects of *Nr4a1* overexpression are extremely complex and may also alter Drd2-mediated function and cholinergic interneuron activity (e.g., via opioidergic stimulation; Ponterio et al., 2013). We did not examine the morphology of MSNs, but, in the hippocampus, Nr4a1 overexpression eliminates neuronal spines (Chen et al., 2014) via transcriptional regulation of cytoskeletal proteins. Nr4a1 also regulates spine density in the striatum (Tian et al., 2010), which impacts Parkinson’s disease and addiction phenotypes (Villalba and Smith, 2013). Finally, Nr4a1 is expressed in glia and is a key regulator of the inflammatory response in microglia and astrocytes (Ipseiz et al., 2014; Rothe et al., 2017; Popichak et al., 2018), another mechanism via which Nr4a1 may impact the response to drugs of abuse (Russo et al., 2009)

In summary, constitutive overexpression (and deletion) of *Nr4a1* in the striatum has profound effects on striosome development and phenotype, and multiple Drd1-related neuronal functions. The exact mechanisms remain to be determined, but the pathways regulated by Nr4a1 may represent novel, druggable approaches to pathologic states associated with increased pERK (e.g., LIDs and cocaine sensitization). In addition, the overexpression of *Nr4a1* in the *Nr4a1*-eGFP mouse and the changes in striatal structure dopaminoceptive function should be taken into account when interpreting data derived from this reporter line.

## Synthesis

Reviewing Editor: Quentin Pittman, University of Calgary Decisions are customarily a result of the Reviewing Editor and the peer reviewers coming together and discussing their recommendations until a consensus is reached. When revisions are invited, a fact-based synthesis statement explaining their decision and outlining what is needed to prepare a revision will be listed below. The following reviewer(s) agreed to reveal their identity: NONE. Note: If this manuscript was transferred from JNeurosci and a decision was made to accept the manuscript without peer review, a brief statement to this effect will instead be what is listed below.

One of the original reviewers of your manuscript and i have examined your revision and response and agree that it is improved. however, a few minor issue remain that you should deal with as follows:

Most of my previous points were satisfactorily addressed. One outstanding issue remains the description of the typical problems with BAC mice. It is not known for D2- ChAT- and Nr4a1-BAC mice whether the overexpression of the reported gene is from the BAC itself (despite the GFP insertion and stop signal) or from abnormal expression from the endogenous locus. Line 377 should be corrected to read “... show increased levels of the gene under study or of other genes encoded on the BAC, either from the BAC itself of from impacts on the endogenous gene locus.” Line 383- is there any evidence of abnormal feed-back mechanisms in the ChATChR2 BAC mice? If not, delete this statement. What is known about the ChATChR2 mice is that they have duplicate copies of VAchT (encoded on the BAC and not followed by a stop signal) and that they strongly overexpress VAchT and, in one report, modestly overexpress ChAT. Line 384- EYFP is misspelled.

In the slice physiology experiments, it is stated that both GFP+ and GFP- neurons within striosome patches were recorded in Nr4a1-BAC mice. Although data is shown in figures, there is no further mention of results from the GFP- neurons in the main text. The

Nr4a1-GFP-positive neurons within the striosomes of Nr4a1-GFP BAC mice have abnormal properties, but whether these are related to abnormal Nr4a1 expression in these neurons, vs. the intrinsic striosomal identity of these neurons remains unclear.

In addition, please replace aka in your abstract with also know as- no English speakers may be puzzled by the abbreviation. Fianlly I note that you have a few photomicrograph panels that lack scale bars; please ensure that each panel of figures includes at least one scale bar.
